# Inequities in maternal postnatal visits among public and private patients: 2004 Pelotas cohort study

**DOI:** 10.1186/1471-2458-9-335

**Published:** 2009-09-14

**Authors:** Alicia Matijasevich, Iná S Santos, Mariângela F Silveira, Marlos R Domingues, Aluísio JD Barros, Paula L Marco, Fernando C Barros

**Affiliations:** 1 Postgraduate Programme in Epidemiology, Federal University of Pelotas, Pelotas, RS, Brazil; 2 Postgraduate Programme in Physical Education, Federal University of Pelotas, Pelotas, RS, Brazil; 3 Postgraduate Programme in Health and Behaviour, Catholic University of Pelotas, Pelotas, RS, Brazil

## Abstract

**Background:**

The postnatal period is the ideal time to deliver interventions to improve the health of both the newborn and the mother. However, postnatal care shows low-level coverage in a large number of countries. The objectives of this study were to: 1) investigate inequities in maternal postnatal visits, 2) examine differences in postnatal care coverage between public and private providers and 3) explore the relationship between the absence of maternal postnatal visits and exclusive breastfeeding, use of contraceptive methods and maternal smoking three months after birth.

**Methods:**

In the calendar year of 2004 a birth cohort study was started in the city of Pelotas, Brazil. Mothers were interviewed soon after delivery and at three months after birth. The absence of postnatal visits was defined as having no consultations between the time of hospital discharge and the third month post-partum. Logistic regression analysis was used to estimate the association between absence of postnatal visits and type of insurance scheme adjusting for potential confounding factors.

**Results:**

Poorer women, black/mixed, those with lower level of education, single mothers, adolescents, multiparae, smokers, women who delivered vaginally and those who were not assisted by a physician were less likely to attend postnatal care. Postnatal visits were also less frequent among women who relied in the public sector than among private patients (72.4% vs 96% among public and private patients, respectively, *x*^2 ^p < 0.001) and this difference was not explained either by maternal characteristics or by health care utilization patterns. Women who attended postnatal visits were more likely to exclusively breastfeed their infants, to use contraceptive methods and to be non-smokers three months after birth.

**Conclusion:**

Postpartum care is available for every woman free of charge in the Brazilian Publicly-funded health care system. However, low levels of postpartum care were seen in the study (77%). Efforts should be made to increase the percentage of women receiving postpartum care, particularly those in socially disadvantaged groups. This could include locally-adapted health education interventions that address women's beliefs and attitudes towards postpartum care. There is a need to monitor postpartum care and collected data should be used to guide policies for health care systems.

## Background

More than a half a million women die every year worldwide from complications in pregnancy and childbirth.[1] Maternal health represents one of the indicators of inequity between rich and poor countries. While the risk of a woman dying from pregnancy or childbirth in the poorest countries can be as high as about 1 in 7, in the richest countries the risk is about 1 in 30,000.[2] Over the last few years, the global community has given greater attention to women's health. The maternal health Millennium Development Goal (MDG) 5 aims to improve maternal health by reducing maternal death and providing universal access to reproductive health care.[3] However, it is believed that MDG 5 is falling severely short of its targets.[4]

As a means of reducing the burden of maternal and newborn deaths, and of the millions of children who die between the ages of 1 month and 5 years, the "Continuum of Care" has recently been introduced into programs focusing on maternal, newborn and child health. This approach is based on the concept that the health and well-being of women, newborns and children are closely linked and should be managed in a unified way.[5] Findings from Tracking Progress in Maternal, Newborn and Child Survival Countdown to 2015 - *The 2008 Report *highlighted priority coverage gaps across the "Continuum of Care" and care during the postnatal period is one of them.[2] There are wide gaps between the proportion of women in the developing world receiving antenatal care (65%) and those who received postnatal care (30%). Postnatal care is frequently missing, even for women who give birth in a health facility. These figures are in contrast to the high coverage of postnatal care in developed countries.[6]

The postnatal period is the ideal time to deliver interventions to improve the health and survival of both the newborn and the mother.[7] Postnatal care helps to identify complications, promote healthy behaviours, ensures the establishment of successful infant feeding, links the mother to family-planning services and the baby to child health care as well as fostering the development of good maternal-infant relationships.[8] However, postnatal care has long been neglected or fragmented and data for postnatal care are either unavailable or show low-level coverage in a large number of countries.[2]

Brazil is a middle-income country with a publicly-funded health care system designed to be integral (offering care for all health problems), universal (covering anyone independent of contribution or employment status) and free (no user fees of any kind). However, one fourth of the population opts for private insurance or - less often - for out-of-pocket payments to private providers. Despite increases in investment in health care provision, maternal mortality rates have remained high in the last decade (74.68 estimated maternal deaths per 100,000 livebirths in 2005).[9] and large social differences in maternal health still persist.[10] Three-quarters of all maternal deaths occurred in the postpartum period. Nevertheless, in Brazil, data regarding coverage of maternal postnatal care is scarce and this critical period is under-researched.[11]

This study aimed to investigate inequities in the absence of maternal postnatal visits among women from the 2004 Pelotas birth cohort study as well as inequities between public and private providers. In addition, we examined the relationship between the absence of maternal postnatal visits and maternal and infant outcomes: exclusive breastfeeding, use of contraceptive methods and smoking assessed at three months after birth.

## Methods

### Research setting and study design

Pelotas is located in Southern Brazil, with a population of about 340,000 inhabitants, 93% of them living in the urban area (2000 Brazilian Demographic Census, IBGE). From 1/1/2004 to 31/12/2004 inclusive, a birth cohort study attempted to enrol all births from mothers resident in the urban area of the city. Eligible subjects for the perinatal study included all livebirths, and stillbirths weighing at least 500 g. When birthweight was not available, all births with at least 20 weeks of gestational age were included in the study. Most deliveries (> 99%) took place in the five hospitals of the city. Births were identified by daily visiting the five maternity hospitals. Mothers were interviewed soon after delivery using a pre-tested structured questionnaire. Information was obtained on demographic, environmental, and socioeconomic variables and on the characteristics of pregnancy, labour, delivery, and healthcare service utilization. Both the interview with the mother and newborn evaluation were carried out within 24 hours of delivery.[12]

At the age of three months, all children in the cohort were sought for the first follow-up visit, with the exception of those known to have died. Mothers were contacted by telephone and invited to schedule an interview either at the Medical School of the Universidade Federal de Pelotas or at home, depending on their preference. For this interview, we defined a seven-day window period, including the day on which the child completed three months and the three days before and after this date (85% of interviews were carried out within this window period). Information about attendance at postnatal visits, breastfeeding patterns, maternal smoking and use of contraceptive methods was assessed.[12]

### Information assessed in the perinatal interview

Family income in the month prior to delivery was collected as a continuous variable (in Reais) and analysed as quintiles. Mother's skin colour was self-reported and categorised as white or black/mixed. The maternal formal education was categorised as: 0-4, 5-8 and ≥ 9 complete school years. Women who were single, widowed, divorced, or lived without a partner were classified as single mothers. Maternal age in complete years was categorised as 12-15, 16-19, 20-34 and ≥ 35 years. Parity was defined as the number of previous viable pregnancies and categorised as 0, 1 and ≥ 2. Smoking habits during pregnancy were based on maternal self-assessment. The smoking variable was constructed taking into account the number of cigarettes smoked in each trimester of pregnancy as well as pregnancy duration and was categorised as never smoked during pregnancy, smoked less than 10 cigarettes per day and smoked 10 or more cigarettes per day during pregnancy. Adequacy of antenatal care was calculated according to the Kessner Index.[13] (which combines the timing of the first prenatal visit with the relation between the total number of visits and pregnancy duration) and categorised as adequate, intermediate, inadequate and no antenatal care. The categories for people assisting the delivery were: physician, student, midwife or nurse and other. Students of midwifery, nursing or medicine were included in the second category. The last category ("other") was used when there was no indication that a health professional or student of any type had been present at delivery. Type of delivery was categorised as vaginal or caesarean section. Type of insurance scheme was categorised as public (government funding through the Unified Health System) or private (private health insurance or out-of-pocket payments to the provider).

### Information assessed at the 3 months follow-up

Postnatal visits were assessed using the following question: "Since your baby was born, have you had a postpartum visit for yourself?" The absence of postnatal visits was defined as having no consultations between the time of hospital and the interview at the 3 months follow-up.

Exclusive breastfeeding at the third month of life, was based on the WHO definition, [14] and referred to as feeding only breast milk to the child, allowing the baby to receive vitamins, minerals or medicine.

Maternal smoking was self-reported. Regular smokers were those women who smoked at least one cigarette per day on an everyday basis.

Information about family planning - whether the woman was using any contraceptive method or not - was collected for all women regardless marital status.

### Data analysis

We restricted our analyses to women who had a liveborn infant who had not required care in a special care or intensive care nursery, who left hospital on the same day as their baby and who had information about postnatal visits in the interview at 3 months.

We used tests for linear trends and *x*^2 ^tests to compare the distribution of absence of postnatal visits by maternal characteristics, health care utilization and type of insurance scheme.

Logistic regression analysis was used to estimate the association between absence of postnatal visits (outcome) and type of insurance scheme adjusting for potential confounding factors. In multivariate analyses, we considered type of insurance scheme as a proximal determinant of lack of postnatal visits, with effects that could be confounded by distal variables. An operational definition of confounding was used, that is, variables that were associated with both the outcome and the predictor of interest, and not part of the causal chain.[15] The decision of which variables to include in the multivariate analysis was based on a conceptual framework describing the postulated hierarchical relationships between exposures.[16] For multivariate analysis, variables were grouped and included in the model on three different levels, using a backward strategy selection. The first level included maternal characteristics such as family income, maternal skin colour, schooling, marital status, age and parity; the second level included smoking during pregnancy and adequacy of antenatal care. Type of delivery, professional assistance at delivery and type of insurance scheme were included in the third level. If the significance level was below 0.20, [17] the variable remained in the model as potential confounders for the next level. Interaction terms between postnatal care and both "type of delivery' and "birth assisted by a doctor" were introduced into the adjusted analysis to assess the potential effect modification. We evaluated the correlation matrix for any evidence of multicollinearity before finalizing the models.

Additional analyses explored the association between exclusive breastfeeding at three months of age, use of contraceptive methods and smoking after birth (each one as outcomes) and the absence of postnatal visits (main exposure). Women without a partner were excluded from the analysis of the association between use of contraceptive methods and the absence of postnatal visits.

All analyses were performed using Stata 10 (StataCorp LP, College Station, Tx). The study protocol was approved by the Medical Ethics Committee of the Federal University of Pelotas, affiliated with the Brazilian Federal Medical Council. Written informed consent was obtained from women who accepted to participate in the study.

## Results

A total of 4,244 mothers were interviewed soon after delivery (losses and refusals in the perinatal phase were below 1%). At the 3^rd ^month follow-up losses and refusals were 4.3%.[12] After excluding women with foetal deaths (n = 56); those women whose newborns required care in a special care nursery or intensive care unit after birth (n = 403); women who left hospital before their newborns or stayed after they were discharged (n = 327); and women without information about postnatal visits (n = 8); 3,497 women remained for analysis. None of the variables included in the analyses had more than 2.5% of missing data.

About four in every five women received postpartum care at public-funded Unified Health System units (80.6%). Private patients were more common among better-off women (58.3% of women in the 5^th ^quintile *vs*. 6.1% of women in the 1^st ^quintile of income), white women (25% of white women *vs*. 11.2% of black/mixed colour women) and women with higher schooling (87.4% of women with ≥ 9 years *vs*. 1.8% of women with ≤ 4 years of formal education). Single mothers (88.4% of single mothers *vs*. 79.1% of women with partner), adolescents (93.6% of adolescents *vs*. 71.6% of women ≥ 35 years old) and multiparae (89.6% of multiparae *vs*. 75.2% primiparae) relied more frequently on the public sector. Women who smoked during pregnancy (91.8% of smokers *vs*. 77.0% of non-smokers) and women with inadequate antenatal care (92.1% of women with inadequate care vs. 65.8% of those with adequate antenatal care) were found more frequently among the public sector. Women from the private sector had higher frequencies of caesarean section and deliveries assisted by a doctor (83.8% *vs*. 34.1% and 99.9% *vs*. 86.4% in the private and public sector). All tests were statistically significant (*x*^2 ^p < 0.001).

**Figure 1:**
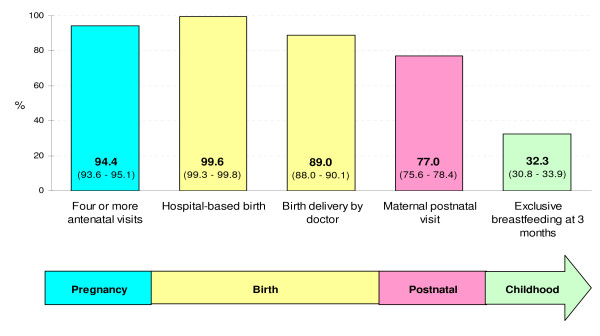
**Prevalence (and 95% confidence intervals) of selected interventions across the continuum of care by insurance scheme, 2004 Pelotas birth cohort study**.

Figure 1 shows the prevalence of selected interventions across the continuum of care in the 2004 Pelotas birth cohort study. Ninety four percent of all women had four or more antenatal care visits (99.9% and 93.1% in the private and public sector, respectively, *x*^2 ^p < 0.001), nearly 100% of all deliveries took place in hospitals (99.9% among private patients and 99.5% among public patients, *x*^2 ^p = 0.244), 89% of women had their babies delivered by a doctor (99.9% and 86.4% in the private and public sector, respectively, *x*^2 ^p < 0.001), 77% of women had at least one postnatal visit (96.0% and 72.4% in the private and public sector, respectively, *x*^2 ^p < 0.001) and nearly 32% of women were exclusively breastfeeding their infants at 3 months after birth (42.7% among private patients and 29.9% among public patients, *x*^2 ^p < 0.001). For most indicators, higher coverage was observed among private patients.

A description of maternal characteristics, health care utilization and type of insurance scheme besides the distribution of women according to absence of postnatal visits are shown in Table 1. The absence of postnatal visits was more frequent among women in the poorest quintiles of family income, among black/mixed women and among those with lower level of education. Single mothers, adolescents, multiparae, smokers and those who did not attend antenatal care were more likely to have had an absence of postnatal visits. Women who delivered by caesarean section, those assisted by a doctor during delivery and those who relied on the private health sector were less likely to have had an absence of postnatal visits compared with women who delivered vaginally, women assisted by a midwife/nurse/student or by no health professional, and women from the public sector, respectively.

**Table 1:** Description of maternal characteristics and type of insurance scheme according to the absence of postnatal visit.

**Variables**	**n**	**Absence of postnatal** **visit** **%**	**p^a^**
Family income (quintiles)			< 0.001^b^
1^st ^(poorest)	691	35.2	
2^nd^	697	30.3	
3^rd^	682	24.1	
4^th^	727	16.9	
5^th ^(better-off)	700	9	
Maternal skin colour			< 0.001
Black/mixed	1272	30.2	
White	2151	18.5	
Maternal schooling (y)			< 0.001^b^
0-4	533	38.5	
5-8	1399	30	
≥ 9	1534	11.6	
Marital status			< 0.001
Single mother	559	30.8	
With partner	2938	21.5	
Maternal age (y)			< 0.001^b^
< 20	656	29.7	
20-34	2374	21.6	
≥ 35	465	20.7	
Parity			< 0.001^b^
0	1372	17.7	
1	925	21.2	
≥ 2	1199	30.4	
Smoking during pregnancy			< 0.001^b^
Never smoked	2620	19.1	
Smoked < 10 cigarettes per day	605	32.4	
Smoked ≥ 10 cigarettes per day	272	39.3	
Adequacy of antenatal care (Kessner Index)			< 0.001^b^
Adequate	1533	12.7	
Intermediate	1065	28	
Inadequate	854	33.4	
No prenatal care	45	57.8	
Type of delivery			< 0.001
Vaginal	1969	28.9	
C-section	1528	15.4	
Professional who assisted the delivery			< 0.001
Doctor	3099	21.4	
Student	318	31.1	
Midwife/nurse	18	44.4	
Nobody/other	46	58.7	
Type of insurance scheme			< 0.001
Public	2813	27.6	
Private	679	4	
Total	3497	23	-

^a ^*x*^2 ^test; ^b ^*x*^2 ^test for trend

The adjusted analysis resembled the crude analysis (Table 2). All variables that were highly associated with absence of postnatal visits in the crude analysis remained associated after adjustment, showing only a reduction in the magnitude of OR's. Postnatal visits were less frequent among the poorest, black/mixed colour women, those who had less education, single mothers, adolescents and multiparae. Women who smoked during pregnancy, those who had not had adequate prenatal care or did not attend at all, women who delivered vaginally and those whose delivery was not assisted by a doctor were also at an increased risk of absence of postnatal visits. Interaction terms between postnatal care and both type of delivery and birth assisted by a doctor were introduced into the adjusted analysis (p = 0.10 and < 0.001, respectively). After controlling for all potential confounders, women in the public sector were less likely to have postnatal visits compared to women who relied on the private sector.

**Table 2:** Crude and adjusted analyses for the association between absence of postnatal visit and maternal characteristics.

**Variables**	**OR crude** **(95% CI)**	**p^a^**	**OR adjusted** **(95% CI)**	**p^a^**
Family income (quintiles)		< 0.001		< 0.001
1^st ^(poorest)	5.48 (4.05; 7.42)		2.61 (1.85; 3.66)	
2^nd^	4.39 (3.24; 5.96)		2.17 (1.55; 3.05)	
3^rd^	3.20 (2.34; 4.38)		2.02 (1.44; 2.82)	
4^th^	2.06 (1.49; 2.85)		1.51 (1.07; 2.13)	
5^th ^(better-off)	Reference		Reference	
Maternal skin colour		< 0.001		< 0.001
Black/mixed	1.91 (1.63; 2.25)		1.37 (1.16; 1.63)	
White	Reference		Reference	
Maternal schooling (y)		< 0.001		< 0.001
0-4	4.76 (3.77. 6.02)		2.64 (2.01; 3.48)	
5-8	3.27 (2.69; 3.97)		2.04 (1.64; 2.54)	
≥ 9	Reference		Reference	
Marital status		< 0.001		< 0.001
Single mother	1.62 (1.33; 1.98)		1.49 (1.20; 1.86)	
With partner	Reference		Reference	
Maternal age (y)		< 0.001		0.001
< 20	1.63 (1.23; 2.15)		1.88 (1.31; 2.70)	
20-34	1.06 (0.83; 1.35)		1.25 (0.95; 1.65)	
≥ 35	Reference		Reference	
Parity		< 0.001		< 0.001
0	Reference		Reference	
1	1.25 (1.01; 1.54)		1.47 (1.15; 1.87)	
≥ 2	2.03 (1.69; 2.45)		1.89 (1.46; 2.43)	
Smoking during pregnancy		< 0.001		< 0.001
Never smoked	Reference		Reference	
Smoked < 10 cigarettes p/day	2.03 (1.67; 2.47)		1.33 (1.03; 1.73)	
Smoked ≥ 10 cigarettes p/day	2.74 (2.11; 3.56)		1.65 (1.32; 2.06)	
Adequacy of antenatal care (Kessner Index)		< 0.001		< 0.001
Adequate				
Intermediate	Reference		Reference	
Inadequate	2.67 (2.18; 3.26)		1.77 (1.42; 2.20)	
No prenatal care	3.44 (2.79; 4.23)		1.95 (1.56; 2.45)	
	9.39 (5.10; 17.29)		3.60 (1.87; 6.92)	
Type of delivery		< 0.001		0.01
Vaginal	2.24 (1.89; 2.65)		1.29 (1.06; 1.57)	
C-section	Reference		Reference	
Professional who assisted the delivery		< 0.001		0.029
Doctor				
Student	Reference		Reference	
Midwife/nurse	1.66 (1.29; 2.14)		1.14 (0.86; 1.50)	
Nobody/other	2.95 (1.16; 7.49)		2.11 (0.77; 5.76)	
	5.23 (2.89; 9.47)		2.35 (1.22; 4.51)	
Type of insurance scheme		< 0.001		< 0.001
Public	9.20 (6.21; 13.64)		3.08 (1.99; 4.79)	
Private	Reference		Reference	

^a ^Wald's test

Exclusive breastfeeding, use of any kind of contraceptive method and maternal smoking, all assessed at three months after birth, were highly associated with postnatal visits in the crude analysis for all women (See additional file 1: Crude and adjusted analyses for the association between postnatal visit and maternal and infant outcomes at the 3^rd ^month follow-up.). After adjustment for potential confounders, women who failed to attend postnatal visits were less likely to breastfeed (although with marginally significance), less likely to use contraceptive methods and more likely to be regular smokers at the third month after birth. The crude and adjusted results in the public sector resembled the results for the whole population. However, among private patients, none of these indicators were associated with absence of postnatal visits, both in the crude and in the adjusted analyses.

## Discussion

This study evaluated inequities in postnatal care in a population enrolled in a birth cohort followed-up from birth to the third month postpartum, from a middle-income country. Poorer women, black/mixed women, those with lower level of education, single mothers, adolescents, multiparae, smokers, women who delivered vaginally and those who were not assisted by a physician were less likely to attend postnatal care. Postnatal visits were also less frequent among women from the public sector and this difference was not explained either by maternal characteristics or by health care utilization patterns. Women who attended postnatal visits were more likely to exclusively breastfeed their infants, to use contraceptive methods and to be non-smokers three months after birth.

The present study aims to fill in a gap in the literature concerning the use of postnatal care according to type of insurance scheme and maternal characteristics. Its strengths include the population-based sample, data collection by standardized questionnaires administered by trained interviewers, high follow-up rates and low missing data (below 3%) for most variables. However, some methodological issues need to be discussed. First, information about postnatal visits was based on maternal recall because care is provided by more than a hundred different facilities, and it would not have been feasible to review medical records. Second, even though traditionally, women have been advised to have a visit in the first weeks after childbirth, timing of postnatal visit was not assessed in our study and, therefore, perhaps a proportion of visits took place later. Thirdly, family income in the month before the birth was used as the marker of socioeconomic status. Around 40% of women worked during pregnancy. Unfortunately, information about maternal work benefits (i.e. paid maternity leave) was not collected in the perinatal questionnaire. Though, it is not possible to know if family income decreased because women stopped working around the time of delivery. Finally, our data are from a single Brazilian city and may not be representative of the rest of the country. However, patterns of postnatal health care use are not routinely assessed and, therefore, comparisons with national averages are not possible yet.

In our study, coverage of five selected interventions (four or more antenatal visits, hospital-based birth, birth delivery by doctor, maternal postnatal visit and exclusive breastfeeding at three months) varied along the "Continuum of Care", with especially low coverage for interventions after birth. The "Countdown to 2015 for maternal, newborn and child survival" initiative reported that coverage of interventions varied widely both between and within countries. Family planning, care in childbirth, postnatal care and case management of illnesses in newborn babies and children showed the lowest coverage among 68 priority countries.[2] In addition, the initiative reported that data for postnatal care were either unavailable or showed poor coverage in most those countries.

Even though there is great variability across and within countries in the reported use of postpartum services, higher levels are found in high-income countries, where less than 10-11% of women do not attend postnatal visits.[18,19] A research based on Demographic and Health Surveys conducted in 30 low-income countries between 1999 and 2004 reported that seven out of ten women do not receive any postpartum care.[6] Figures were worst for countries like Ethiopia where 90% of women did not receive any postpartum care, followed by Bangladesh (73%), Nepal (72%) and Rwanda (71%). Other countries showed substantial proportions of women who did not receive any postpartum care, including Burkina Faso (44%), Cambodia (46%), Haiti (55%), Kenya (46%) Malawi (41%), Mali (49%), Nigeria (465), Uganda (57%) and Zambia (41%). On average, in the 30 countries examined, nearly 40 percent of women did not receive a postpartum care check-up. [6]

Information about postnatal care in Brazil is scarce; however, two studies were identified. One study, from the State of Ceará, Northeast Brazil, showed that among the 207 women interviewed, the vast majority (98.1%) reported having received no postpartum care at all.[20] The other study, from the city of Pelotas, carried out ten years ago, showed that out of 95 mothers studied, 25 (25.5%) did not attend postpartum visits.[21]

In our study, higher rates of absent postnatal visits were found in certain population subgroups, such as less educated women and those who had received none or inadequate antenatal care, findings that have been reported consistently in high-, [19] middle- and low-income countries.[21-23] In agreement with previous investigations, women living in disadvantaged economical conditions.[22-24] are at high risk of not receiving postnatal care.

Poor women are more likely to suffer complications from pregnancy and childbirth, [25] which in turn could make them more likely to become poorer perpetuating the vicious circle of poverty, which is very hard to break. Women without a partner,[19,22] adolescents,[22,23] multiparae,[19] women who smoked during pregnancy,[19] and those who had non-medical birth attendance.[26] were at higher risk of absent postnatal visits. Our finding that women who delivered vaginally had a higher prevalence of absent postnatal visits was supported by one previous research.[23] but not by others. [19,21]

In Brazil, postpartum care is available for every woman free of charge in the Brazilian Publicly-funded health care system. However, 19% of women opted to have their postnatal care services covered by private insurance or by out-of-pocket payments to private providers. After birth, women are routinely informed to seek for postnatal services in the first weeks after delivery. Unlike other countries where community-based postnatal care is available, [8] in Brazil, postnatal care is not provided at home. However, the city counts on more than a hundred facilities where postnatal care is available.

Differences in postnatal care were found between private and public patients. While postnatal care coverage among private patients was almost universal (96%), about one out of four women from the public sector (28%) reported absence of postnatal visit. This finding was also reported in other study.[23] Since in Brazil there are no fees for the public sector users, nor other direct out-of-pocket payments, the absence of postnatal visits is likely to be related to other types of economic barriers (e.g. transportations costs, or time lost from work), or alternatively by cultural or educational constrains. In rural Tanzania, postnatal services are perceived to be important and are routinely provided. However, unless there is a serious issue related to maternal complications, these services target the child and little attention is paid to the mother.[27] Previous analyses showed that children from the 2004 Pelotas birth cohort attended more than 10 preventive medical consultations in their first year of life,[28] which suggests that opportunities for maternal postnatal visits are being missed and that the comprehensiveness of care needs to be improved in the public health system. Among Palestinian women, the most frequent reason for not obtaining postnatal care was their lack of perceived need (especially if they were feeling well), followed by not having been told by health care providers to obtain postnatal care.[23] Women's knowledge on the importance of postnatal care and perceptions about the quality of health care available are also known factors that influence postnatal care attendance.[29]

Postnatal care provides the opportunity to effectively reduce deaths of mothers and neonates, to support the adoption of healthy behaviours, to begin preconception or family-planning counselling, to increase breastfeeding rates, to improve maternal physical and emotional wellbeing and to decrease morbidities and disabilities of the mothers and their infants.[8] Even though in our study, the content or quality of the postnatal visits was not specifically investigated, women who attended postnatal care were more likely to exclusively breastfeed their infants, to use any kind of contraceptive method and to be non-smokers at three months after birth, showing that postpartum care could make a difference to the lifestyle of women and their infants. However, the possibility of reverse causality cannot not be ignored.

Our study lacks information about the timing of postnatal care and the types of procedures that were carried out. Worldwide there is controversy concerning the timing of postpartum care and its capacity to meet women's health needs after childbirth. Recently, some investigators have questioned the necessity for the examinations traditionally seen as the central component of the consultation.[18] However, there is consensus that certain aspects of postnatal care (such as the timing and location, i.e. at home or in a health care facility) need further research and translation into guidelines and protocols.[2]

Even though the analysis of caesarean sections is not the focus of this paper, it is worth noting that Brazil presents one of the highest rates of caesarean sections in the world. [30] In the city of Pelotas, between 1982 and 2004, rates of caesarean section increased from 28% to 43%, reaching more than 80% in the private sector. [31] Twenty years ago, the World Health Organization recommended that no more than 15% of deliveries should be delivered by caesarean section.[32] Higher levels of caesarean sections do not necessarily indicate better perinatal care and have not proved benefit either for the mothers or for their offspring. Moreover, an increase in the rate of caesarean delivery was associated with an increase in foetal mortality rates and higher numbers of babies admitted to intensive care for seven days or longer even after adjustment for preterm delivery.[33]

## Conclusion

Differences in the absence of postnatal visits according to women's characteristics and type of health provider were found in the study. Higher coverage, almost universal, was found in the private sector. In terms of policy implications, our findings suggest the need for investing in the demand and supply areas. Understanding who is at risk for not receiving postnatal care is the first step in developing targeted messages for women and health professionals to assess how different social groups are benefiting from the progress of health care. Efforts should be made to increase the percentage of women receiving postpartum care, particularly those in the socially disadvantaged groups, including locally tailored health education interventions that address women's beliefs and attitudes towards postpartum care. There is a need to monitor postpartum care and collected data should be used to guide policies for health care systems. Future research needs to focus on quality of postnatal care, ensuring that timing and content match the needs of women and their children.

## Competing interests

The authors declare that they have no competing interests.

## Authors' contributions

AM identified the research question, conducted the analyses and wrote the first draft of the article. ISS contributed to the interpretation of the findings and the writing of the article. MFS, MRD, PLM, AJDB and FCB contributed to the interpretation of the analysis and assisted with the editing of the article. All authors read and approved the final version of the manuscript.

## Pre-publication history

The pre-publication history for this paper can be accessed here:



## Supplementary Material

Additional File 1 **Crude and adjusted analyses for the association between postnatal visit and maternal and infant outcomes at the 3^rd ^month follow-up.** Click here for file

## References

[B1] AbouZahr C (2003). Global burden of maternal death and disability. British medical bulletin.

[B2] Bryce J, Daelmans B, Dwivedi A, Fauveau V, Lawn JE, Mason E, Newby H, Shankar A, Starrs A, Wardlaw T (2008). Countdown to 2015 for maternal, newborn, and child survival: the 2008 report on tracking coverage of interventions. Lancet.

[B3] United Nations General Assembly (2001). Road Map Toward the Implementation of the United Nations Millennium Declaration: Report of the Secretary General.

[B4] Millennium Development Goal 5: Improve maternal health. http://www.dfid.gov.uk/mdg/.

[B5] Kerber KJ, de Graft-Johnson JE, Bhutta ZA, Okong P, Starrs A, Lawn JE (2007). Continuum of care for maternal, newborn, and child health: from slogan to service delivery. Lancet.

[B6] Fort AL, Kothari MT, Abderrahim N (2006). Postpartum Care: Levels and Determinants in Developing Countries. DHS Comparative Reports No 15.

[B7] Finger WR (1997). Better postpartum care saves lives. Network.

[B8] MacArthur C (1999). What does postnatal care do for women's health?. Lancet.

[B9] Taxa de mortalidade materna. http://tabnet.datasus.gov.br/cgi/idb2007/c03.htm.

[B10] Diaz MD (2002). Socio-economic health inequalities in Brazil: gender and age effects. Health economics.

[B11] Diniz SG, Bick D, Bastos MH, Riesco ML (2007). Empowering women in Brazil. Lancet.

[B12] Barros AJ, da Silva dos Santos I, Victora CG, Albernaz EP, Domingues MR, Timm IK, Matijasevich A, Bertoldi AD, Barros FC (2006). [The 2004 Pelotas birth cohort: methods and description]. Revista de saude publica.

[B13] Institute of Medicine (1974). Infant deaths, an analysis by maternal risk and health care. Contrasts in health status, based on: The American College of Obstetricians and Gynecologists: Standards for Obstetric-Gynecologic Services.

[B14] World Health Organization (2004). Promoting proper feeding for infants and young children.

[B15] Rothman KJ, Greenland S, Rothman KJ, Greenland S (1998). Precision and validity in epidemiologic studies. Modern Epidemiology.

[B16] Victora CG, Huttly SR, Fuchs SC, Olinto MT (1997). The role of conceptual frameworks in epidemiological analysis: a hierarchical approach. Int J Epidemiol.

[B17] Maldonado G, Greenland S (1993). Simulation study of confounder-selection strategies. American journal of epidemiology.

[B18] Bick DE, MacArthur C (1995). Attendance, content and relevance of the six week postnatal examination. Midwifery.

[B19] (2007). Postpartum care visits--11 states and New York City, 2004. Mmwr.

[B20] Lindsay AC, Dubowitz T, Andrade FM, Campos JS, Peterson KE (2007). Maternal Health Care Services in the State of Ceará, Northeast Brazil. Women's Health and Urban Life.

[B21] Bertoni A, Santos I (2000). [Predictive factors of non-compliance with puerperal review among mother at primary health care facilities in Pelotas, Brazil]. Revista AMRIGS, Porto Alegre.

[B22] Letamo G, Rakgoasi SD (2003). Factors associated with non-use of maternal health services in Botswana. Journal of health, population, and nutrition.

[B23] Dhaher E, Mikolajczyk RT, Maxwell AE, Kramer A (2008). Factors associated with lack of postnatal care among Palestinian women: a cross-sectional study of three clinics in the West Bank. BMC pregnancy and childbirth.

[B24] Anson O, Sun S (2004). Health inequalities in rural China: evidence from HeBei Province. Health & place.

[B25] The State of the World's Children - 2009 - Maternal and newborn health. http://www.unicef.org/publications/files/SOWC_2009_Main__Report__03112009.pdf.

[B26] Hove I, Siziya S, Katito C, Tshimanga M (1999). Prevalence and associated factors for non-utilisation of postnatal care services: population-based study in Kuwadzana peri-urban area, Zvimba district of Mashonaland West Province, Zimbabwe. JSTR: African Journal of Reproductive Health.

[B27] Mrisho M, Obrist B, Armstrong-Schellenberg J, Haws RA, Mushi AK, Mshinda H, Tanner M, Schellenberg D (2009). The use of antenatal and postnatal care: perspectives and experiences of women and health care providers in rural southern Tanzania. BMC pregnancy and childbirth.

[B28] Cesar JA, Matijasevich A, Santos IS, Barros AJ, Dias-da-Costa JS, Barros FC, Victora CG (2008). The use of maternal and child health services in three population-based cohorts in Southern Brazil, 1982-2004. Cadernos de saude publica/Ministerio da Saude, Fundacao Oswaldo Cruz, Escola Nacional de Saude Publica.

[B29] Timyan J, Brechin SHG, Measham DM, Ogunleye B, Koblinsy MTK, Gay J (1993). Access to care: more than a problem of distance. The Health of Women: A Global Perspective.

[B30] Ministério da Saúde (2004). Saúde Brasil 2004. Uma análise da situação de saúde.

[B31] Barros FC, Victora CG, Barros AJ, Santos IS, Albernaz E, Matijasevich A, Domingues MR, Sclowitz IK, Hallal PC, Silveira MF (1982). The challenge of reducing neonatal mortality in middle-income countries: findings from three Brazilian birth cohorts in 1993, and 2004. Lancet.

[B32] World Health Organization (1985). Appropriate technology for birth. Lancet.

[B33] Villar J, Valladares E, Wojdyla D, Zavaleta N, Carroli G, Velazco A, Shah A, Campodonico L, Bataglia V, Faundes A (2006). Caesarean delivery rates and pregnancy outcomes: the 2005 WHO global survey on maternal and perinatal health in Latin America. Lancet.

