# Supplementary material for: Inequities in maternal postnatal visits among public and private patients: 2004 Pelotas cohort study
Source: BMC Public Health. 2009 Sep 14;9:335. doi: 10.1186/1471-2458-9-335 (PMC2749044; doi:10.1186/1471-2458-9-335)
Supplement: Additional File 1 — Crude and adjusted analyses for the association between postnatal visit and maternal and infant outcomes at the 3rd month follow-up. [file 1471-2458-9-335-S1.doc]

Table 3. Crude and adjusted analyses for the association between exclusive breastfeeding, use of any kind of contraceptive method and maternal smoking after birth and absence of maternal postnatal visit by type of insurance scheme, 2004 Pelotas birth cohort study.

| Variables^*^ | n | Absence of postnatal visit  % | OR crude  (95% CI) | p^**^ | OR adjusted  (95% CI) | p^**^ |
| --- | --- | --- | --- | --- | --- | --- |
| ***All women*** | | | | | | |
| Exclusive breastfeeding  No  Yes | 2270  1085 | 25.4  17.1 | 1.66 (1.38; 1.99)  Reference | <0.001 | 1.21 (0.99; 1.48) ^a^  Reference | 0.058 |
| Use of any kind of contraceptive method ***  No  Yes | 212  2725 | 43.4  19.8 | 3.10 (2.33; 4.14)  Reference | <0.001 | 2.70 (1.97; 3.70) ^b^  Reference | <0.001 |
| Maternal smoking  Yes  No | 907  2589 | 35.0  18.8 | 2.32 (1.96; 2.74)  Reference | <0.001 | 1.36 (1.02; 1.82) ^c^  Reference | 0.037 |
| ***Private sector*** | | | | | | |
| Exclusive breastfeeding  No  Yes | 371  276 | 4.6  3.3 | 1.42 (0.63; 3.25)  Reference | 0.400 | 1.41 (0.60; 3.30) ^d^  Reference | 0.432 |
| Use of any kind of contraceptive method ***  No  Yes | 26  588 | 3.9  3.1 | 1.27 (0.16; 9.87)  Reference | 0.821 | 1.20 (0.15; 9.43) ^e^  Reference | 0.865 |
| Maternal smoking  Yes  No | 70  608 | 5.7  3.8 | 1.54 (0.52; 4.59)  Reference | 0.437 | 2.00 (0.44; 9.12) ^f^  Reference | 0.373 |
| ***Public sector*** | | | | | | |
| Exclusive breastfeeding  No  Yes | 1896  808 | 29.5  21.8 | 1.50 (1.24; 1.82)  Reference | <0.001 | 1.20 (0.98; 1.48) ^g^  Reference | 0.076 |
| Use of any kind of contraceptive method ***  No  Yes | 185  2133 | 49.2  24.4 | 3.00 (2.21; 4.06)  Reference | <0.001 | 2.83 (2.04; 3.92) ^h^  Reference | <0.001 |
| Maternal smoking  Yes  No | 833  1980 | 37.5  23.4 | 1.96 (1.64; 2.33)  Reference | <0.001 | 1.35 (1.00; 1.82) ^i^  Reference | 0.049 |

^*^ variables assessed at the 3^rd^ month follow-up; ^**^ Wald test; *** women without partner were excluded from this analysis

^a^ adjusted for income, schooling, marital status, parity, smoking during pregnancy and adequacy of antenatal care

^b^ adjusted for maternal skin color, schooling, age, parity, adequacy of antenatal care and type of delivery

^c^ adjusted for income, maternal skin color and smoking during pregnancy

^d^ adjusted for maternal skin color, parity, smoking during pregnancy and type of delivery

^e^ adjusted for maternal skin color

^f^ adjusted for smoking during pregnancy

^g^ adjusted for income, schooling, marital status, parity, smoking during pregnancy and adequacy of antenatal care

^h^ adjusted for maternal skin color, schooling, age, parity, adequacy of antenatal care and type of delivery

^i^ adjusted for maternal skin color, age, smoking during pregnancy and type of delivery
